# Scapholunate advanced collapse: a pictorial review

**DOI:** 10.1007/s13244-014-0337-1

**Published:** 2014-06-03

**Authors:** Brian T. Tischler, Luis E. Diaz, Akira M. Murakami, Frank W. Roemer, Ajay R. Goud, William F. Arndt, Ali Guermazi

**Affiliations:** 1 Department of Radiology, Boston Medical Center, Boston University School of Medicine, 820 Harrison Avenue, FGH Building, 3rd Floor, Boston, MA 02118 USA; 2 Department of Radiology, VA Healthcare System, West Roxbury, MA 02132 USA; 3 Department of Radiology, University of Erlangen, Erlangen, Germany

**Keywords:** Scapholunate advanced collapse, Arthritis, Wrist, Radiography, Review

## Abstract

Scapholunate advanced collapse (SLAC) is the most common cause of osteoarthritis involving the wrist. Along with clinical investigation, radiological studies play a vital role in the diagnosis of SLAC wrist. Given that the osteoarthritic changes that are seen with SLAC occur in a predictable progressive pattern, it is important to understand the pathological evolution of SLAC to be able to recognise the associated progressive imaging findings seen with this disease process. Focusing on radiological findings, this article provides a pictorial review of the anatomy of the scapholunate interosseous ligament as well as the common terminology and biomechanical alterations seen in the pathway leading to the development of SLAC arthropathy. We will then discuss two additional common causes of SLAC wrist and their imaging findings, namely scaphoid non-union advanced collapse and calcium pyrophosphate dehydrate disease. In addition, we will provide a brief overview of the current treatment options of these pathological entities.

• *SLAC is the most common cause of osteoarthritis involving the wrist.*

• *Arthritic changes of SLAC occur in a predictable progressive pathological and radiographic pattern.*

• *Imaging is key for diagnosing, monitoring progression and assessing post-treatment changes of SLAC.*

## Introduction

Osteoarthritis of the wrist is a painful disease process, which can lead to decreased function and disability of the upper extremity. Scapholunate advanced collapse (SLAC) is a frequently encountered progressive form of wrist osteoarthritis that most often occurs secondary to traumatic injury of the scapholunate ligament [1]. Other causes of SLAC include calcium pyrophosphate dehydrate (CPPD) crystal deposition disease [2], scapholunate non-union advanced collapse (SNAC), idiopathic avascular necrosis of the scaphoid (Preiser disease), midcarpal instability, intra-articular fractures involving the radioscaphoid or capitolunate joint, perilunate dislocation and Kienbock’s disease [3, 4]. Imaging, including radiography, computed tomography (CT) and magnetic resonance imaging (MRI), is frequently utilised to diagnose SLAC, monitor its progression and to assess post-treatment changes. This article will provide a pictorial review of the relevant anatomy, terminology, pathological processes, common causes and treatment options pertinent to the understanding of SLAC wrist.

## Scapholunate interosseous ligament

**Fig. 1:**
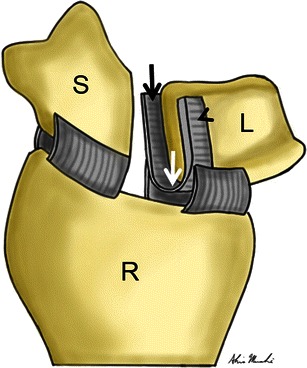
Illustration of scapholunate interosseous ligament anatomy. The drawing depicts a slightly oblique, coronal view of the distal radius (*R*), scaphoid (*S*) and lunate (*L*). The scapholunate ligament has been transected to demonstrate its three distinct parts, which include the dorsal (*arrowhead*), membranous (*white arrow*) and volar (*black arrow*) components. Note that the dorsal component is the thickest

The scapholunate interosseous ligament binds the scaphoid and lunate together, and is the primary stabilising ligament between these two bones. The scaphotrapezium and radioscaphocapitate ligaments are secondary stabilisers of the scapholunate articulation, and therefore are not as vital as the scapholunate interosseous ligament in stabilising the scapholunate articulation [5]. The scapholunate ligament is composed of three distinct parts, which include the dorsal, membranous and volar components (Fig. 1). Even though the volar and membranous components are important for scapholunate stability, the dorsal component of the scapholunate ligament is the thickest and strongest, and therefore plays the most crucial role in stabilisation [6].

## Terminology in the pathway to the development of SLAC wrist

There are four commonly used terms used to describe the pathology seen in the pathway to the development of SLAC wrist. These terms are scapholunate dissociation, scapholunate diastasis, rotary subluxation of the scaphoid and dorsal intercalated segment instability (DISI). These pathological entities can be identified on imaging by measuring specific angles and distances between the different bones of the wrist (Table 1).

**Table 1:** Key radiological measurements of the SLAC wrist pathological terms

Scapholunate diastasis (PA radiograph)	Rotary subluxation of the scaphoid (lateral radiograph)	Dorsal intercalated segment instability (DISI) (lateral radiograph)
1. Scapholunate interval >4 mm (2–4 mm is suspected scapholunate diastasis)	1. Scapholunate angle >60–80° (scaphoid tilted volarly)	1. Scapholunate angle >80° (60–80° is suspected DISI; lunate tilted dorsally)
	2. Radioscaphoid angle >60°	2. Radiolunate angle >10°
		3. Capitolunate angle >30°

Scapholunate dissociation is the loss of synchronous motion or normal alignment between the scaphoid and lunate bones usually from ligamentous injury [7, 8]. The mechanism of injury in scapholunate dissociation is most commonly trauma causing wrist extension, ulnar deviation and intercarpal supination [9]. Eventually scapholunate dissociation leads to misalignment of other scaphoid joints and ultimately to osteoarthritis (SLAC wrist) [8].

Scapholunate diastasis is the term used to describe an abnormal increase in the scapholunate interval. Scapholunate diastasis occurs when there is a functionally complete tear of the scapholunate ligament. Scapholunate diastasis can be seen in the setting of scapholunate dissociation. However, scapholunate dissociation and scapholunate diastasis are not truly synonyms as one may have dissociation with a preserved width of the scapholunate interval [8].

**Fig. 2:**
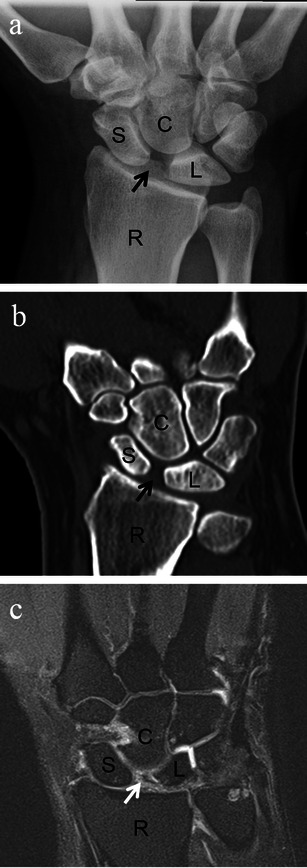
Scapholunate dissociation. Posteroanterior radiograph (**a**) and coronal CT reformat image (**b**) of the wrist demonstrating widening (diastasis) of the scapholunate interval (*black arrow*, **a**, **b**) of approximately 5 mm in a patient with wrist pain and history of prior trauma. **c** Coronal STIR MRI image of the wrist in a different patient shows in addition to scapholunate widening, complete tear of the scapholunate ligament (*white arrow*). *S* scaphoid, *L* lunate, *C* capitate, *R* radius

The imaging findings which can be seen with scapholunate dissociation include scapholunate diastasis, which is described as having a scapholunate distance greater than 4 mm as measured at the midpoint of the scapholunate joint on the posteroanterior view, or having double the width of the scapholunate interval compared with a normal capitolunate joint interval [8]. Scapholunate dissociation may be suspected when the scapholunate interval is 2–4 mm [10]. Widening of the scapholunate interval on imaging has been called the “Terry Thomas sign”, referring to the famous actor with a prominent gap in his front teeth [11]. MRI is useful in that in addition to diastasis, it may also directly show a scapholunate ligament tear (Fig. 2). Ultrasound can be used as a low cost alternative to MRI to identify scapholunate ligament tears. Ultrasound is particularly useful for assessing patients with prior wrist fractures with subsequent surgical fixation hardware placement because the hardware can cause metal susceptibility artefact on MRI, limiting the ability to evaluate the wrist ligaments [12].

**Fig. 3:**
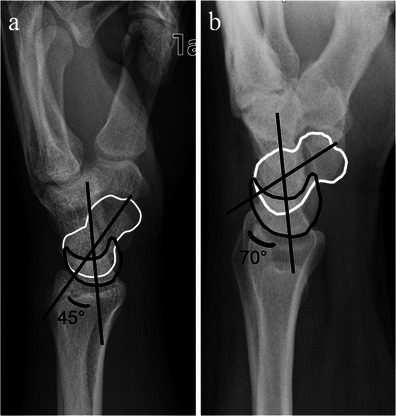
Normal alignment versus rotary subluxation of the scaphoid. **a** Lateral radiograph of the wrist showing normal alignment and rotation of the lunate (*black outline*) and scaphoid (*white outline*), with a normal scapholunate angle of 45° (*black lines*). **b** Lateral radiograph of the wrist of a different patient depicting rotary subluxation of the scaphoid. There is abnormal rotation of the scaphoid (*white outline*) where the proximal pole of the scaphoid has moved dorsally and there is volar tilt, causing an abnormal scapholunate angle of 70° (*black lines*). Note is made that the normal lunate angulation is maintained

Rotary subluxation of the scaphoid is another term commonly encountered in the discussion of the pathway to the development of SLAC wrist. Rotary subluxation of the scaphoid is a pathological displacement of the scaphoid with or without apparent scapholunate disruption secondary to ligamentous injury. Rotation most commonly occurs with the proximal pole of the scaphoid moving dorsally and rotates onto the dorsal aspect of the radius and capitate [8] (Fig. 3). This particular pattern of scaphoid rotation has been demonstrated to occur when there is interruption of both the dorsal radiocarpal ligament and the scapholunate interosseous ligament [7]. However the scaphoid can also rotate in other directions depending on different forms of ligamentous injury [8].

**Fig. 4:**
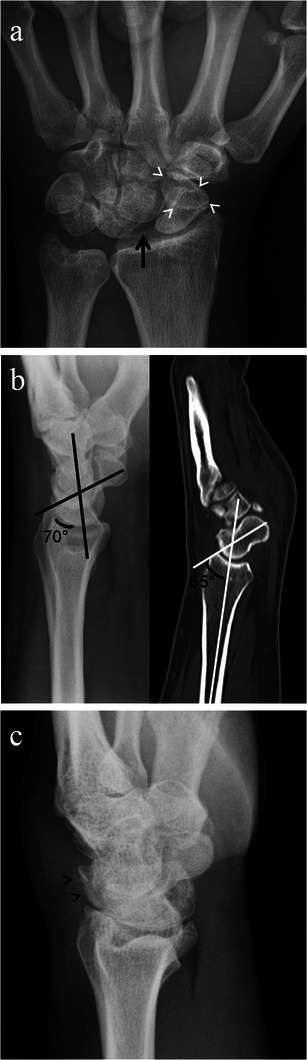
Rotary subluxation of the scaphoid. Imaging findings in three different patients. **a** Posteroanterior radiograph of the wrist shows a widened scapholunate distance (*black arrow*) and foreshortening of the scaphoid with a “signet ring” sign (*white arrowheads*). **b** Lateral radiograph (*left image*) and sagittal CT reformat image (*right image*) of the wrist demonstrating an increased scapholunate angle of 70° on radiograph (*black lines*) and increased radioscaphoid angle of 65° on CT (*white lines*). **c** Lateral wrist radiograph showing subluxation of the scaphoid onto the dorsal rim of the radius (*black arrowheads*)

There are several imaging findings that can be observed with rotary subluxation of the scaphoid. On the PA radiograph, scapholunate interval widening (scapholunate diastasis) may be demonstrated and/or foreshortening of the scaphoid can be seen, causing the “signet ring” sign (produced by the scaphoid tubercle being superimposed on the waist). On the lateral radiograph, an increased radioscaphoid angle >60° and/or a scapholunate angle >60–80° with a normal radiolunate angle can be seen [8]. The normal scapholunate angle ranges from 30 to 60° [7]. Subluxation of the scaphoid onto the dorsal rim of the radius can also sometimes be demonstrated on the lateral radiograph (Fig. 4).

**Fig. 5:**
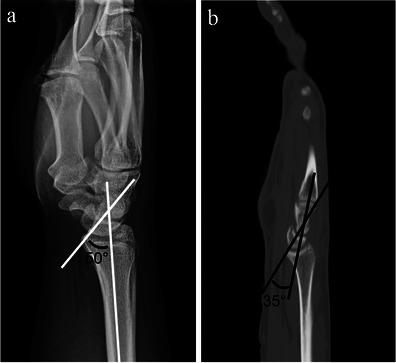
DISI. **a** Lateral wrist radiograph demonstrating dorsal tilt of the lunate with an increased radiolunate angle of 50° (*white lines*). Note is made of concomitant increased volar angulation of the scaphoid and an increased scapholunate angle. **b** Sagittal CT reformat image of the wrist of a different patient demonstrating dorsal tilt of the lunate with an increased capitolunate angle of 35° (*black lines*)

DISI frequently occurs concomitantly with rotary subluxation of the scaphoid, due to alteration of the normal scapholunate articulation. It is most commonly due to ligamentous injury of the scapholunate interosseous ligament or to scaphoid non-union advanced collapse. In DISI there is dorsal angulation of the lunate as it rotates about its articulation with the radius. On the lateral wrist radiograph, DISI demonstrates dorsal tilt of the lunate with a radiolunate angle >10°, capitolunate angle >30° and/or a scapholunate angle >80° (60–80° is suspected DISI) [13] (Fig. 5). On the frontal radiograph scapholunate diastasis may be present. As opposed to DISI, volar intercalated segment instability (VISI) occurs due to injury of the lunotriquetral interosseous ligament causing volar tilt of the lunate. VISI is not associated with scapholunate dissociation, rotary subluxation of the scaphoid or SLAC osteoarthropathy.

It is controversial whether scapholunate dissociation and rotary subluxation of the scaphoid (as well as often concomitantly occurring DISI) are the same terms, or if one of these is a subset of the other. However, it seems that there are subtle but definite differences between these terms as rotary subluxation of the scaphoid and DISI focus on the individual orientation of the scaphoid and lunate, respectively, while scapholunate dissociation focuses on the relationship between the scaphoid and lunate [8].

## SLAC wrist pathogenesis

Osteoarthritis of the wrist occurs almost exclusively (95 %) as a periscaphoid problem. There are three different patterns of arthropathy seen about the wrist which include SLAC, triscaphe arthritis (between the trapezium, trapezoid and distal scaphoid) and a combination pattern. SLAC wrist is the most common type of wrist arthritis and accounts for approximately 55 % of all wrist arthritis [14].

SLAC is an osteoarthropathy of the carpus secondary to altered stress around an unstable scaphoid. In SLAC, there is ligamentous instability of the wrist including disruption of the scapholunate ligament, which causes scapholunate dissociation, scapholunate diastasis, rotary subluxation of the scaphoid and DISI. Changes in alignment of the carpus, particularly changes in position of the scaphoid, result in inordinate stress and load to be placed predominantly on the radioscaphoid and capitolunate joints.

**Fig. 6:**
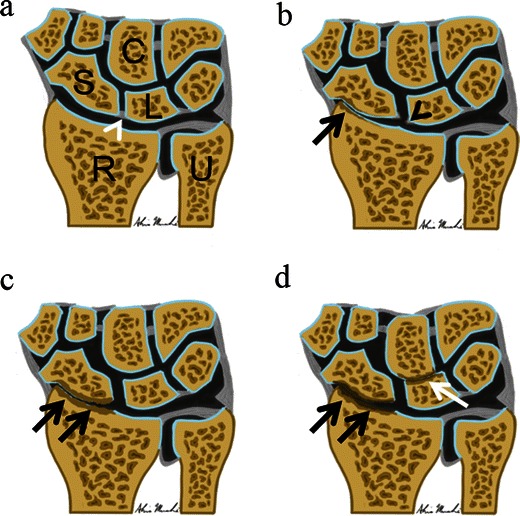
Coronal illustrations of the wrist depicting the progressive osteoarthritic changes of SLAC arthropathy. **a** Normal wrist demonstrating an intact scapholunate ligament (*white arrowhead*), normal scapholunate interval and preservation of the joint spaces. (*S* scaphoid, *L* lunate, *C* capitate, *R* radius, *U* ulna). **b** Stage I: Early findings of SLAC arthropathy including scapholunate ligament tear (*black arrowhead*) with mild widening of the scapholunate interval, as well as early osteoarthritic changes involving the most radial portion of the radioscaphoid joint (*black arrow*). **c** Stage II: Progression of SLAC arthropathy with worsening osteoarthritic changes which now involve the entire radioscaphoid articulation (*black arrows*), and there has been increased widening of the scapholunate interval. **d** Stage III: Further progression of SLAC arthropathy depicted by worsening radioscaphoid joint osteoarthritic changes (*black arrows*), and there is now narrowing of the capitolunate joint space with associated osteoarthritic changes (*white arrow*). This will eventually progress to further proximal migration of the capitate

The sequence of events and mechanical alterations seen in SLAC wrist are sequential and consistent with a predictable progressive pattern of involvement. Three stages of SLAC arthropathy have been described: where osteoarthritic changes initially occur at the most radial aspect of the radioscaphoid joint (Stage I), progressing to involve the entire radioscaphoid joint (Stage II) and eventually affect the capitolunate joint (stage III), with sparing of the radiolunate joint until very late in the arthritic disease process (Fig. 6). Eventually more pronounced separation between the proximal pole of the scaphoid and the lunate occurs. This allows the capitate to migrate proximally, displacing the lunate ulnarward culminating with the SLAC pattern of osteoarthropathy [8, 14, 15].

The most common cause of SLAC is rotary subluxation of the scaphoid, which can be attributed to the elliptical configuration of the radioscaphoid joint. Even with advanced cases of SLAC, the articulation between the lunate and radius is grossly preserved due to the spherical nature of this joint, as opposed to the elliptical nature of the radioscaphoid joint [4, 16, 17].

**Fig. 7:**
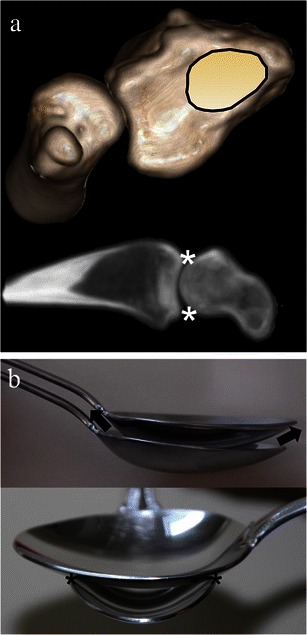
Elliptical configuration of the radioscaphoid joint. **a** Upper image is a 3D volume rendered image of a normal CT of the wrist which depicts the distal ulna and radius viewed en face, showing the elliptical nature of the radial fossa’s scaphoid articulation site (*black outline*). Lower image depicts a sagittal CT reformat view of the radioscaphoid joint. Due to the elliptical nature of the radioscaphoid articulation, changes in position of the scaphoid in relation to the carpus due to rotary subluxation of the scaphoid, will result in incongruence with excessive loading and contact at the dorsal and volar aspects of the radioscaphoid joint (*white asterisks*). **b** The normal radioscaphoid joint resembles two parallel nested spoons as depicted by the upper image. With flexion and extension (*arrows*) full articular contact occurs between the surfaces with congruence and even distribution of the load throughout the joint. Lower image depicts what occurs with rotary subluxation of the scaphoid. When the axis of the spoons changes and they are now perpendicular to each other, the contact surfaces are disrupted and the load is shifted to the dorsal and volar aspect of the articulation leading to destruction and degeneration (*black asterisks*)

Due to the elliptical configuration of the radioscaphoid articulation, pathological changes in position of the scaphoid in relationship to the remaining carpus results in incongruence, excessive loading and contact at the dorsal and volar aspects of the radioscaphoid joint. The normal radioscaphoid joint can be envisioned to resemble two nested spoons which sit parallel on top of one another. With flexion and extension of the normal wrist, full articular contact occurs between the radioscaphoid joint surfaces with congruence and evenly distributed load throughout the joint. If the axis of the spoons changes and they are now perpendicular to each other as one may think happens with even minor rotation of the scaphoid (rotary subluxation), then the contact surfaces are disrupted and the load is shifted to the dorsal and volar aspects of the radioscaphoid articulation leading to destruction and degeneration [4, 16, 17] (Fig. 7).

**Fig. 8:**
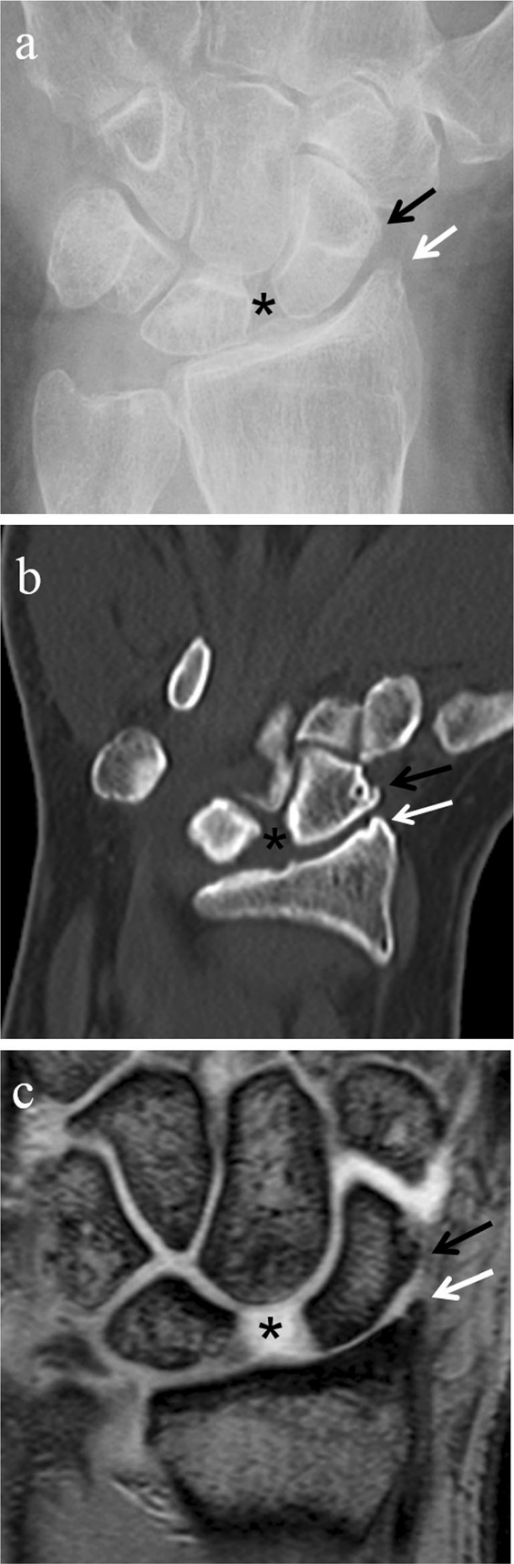
SLAC wrist early radiological findings (Stage I). Posteroanterior radiograph of the wrist (**a**), coronal CT reformat image of the wrist (**b**) and coronal proton density fat suppressed MRI image of the wrist (**c**) in three different patients show spurring at the articular/non-articular junction on the radial side of the scaphoid (*black arrows*) and at the radial styloid tip (*white arrows*), with loss of the normal rounded curvature of the radial styloid. Scapholunate diastasis is seen (*black asterisks*)

**Fig. 9:**
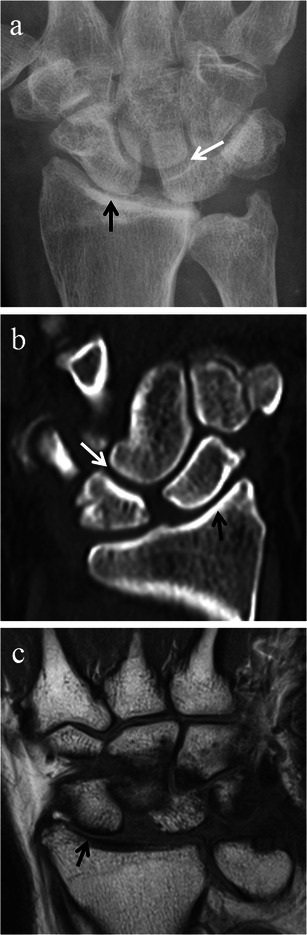
SLAC wrist radiological progression (Stage II). Posteroanterior radiograph (**a**), coronal CT reformat image (**b**) and coronal T1-weighted MRI image of the wrists (**c**) of three different patients, demonstrating narrowing of the radioscaphoid articulation (*black arrows*) in addition to radial styloid and scaphoid spurring. Note the preservation of the capitolunate joint at this point (*white arrows*)

**Fig. 10:**
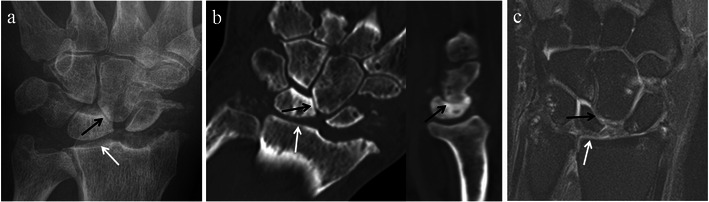
SLAC wrist with further radiological progression (Stage III). Posteroanterior radiograph (**a**) and coronal and sagittal CT reformat images (**b**) of the same patient, as well as a coronal STIR MRI image of the wrist (**c**) in a different patient with SLAC wrist arthropathy. Images demonstrate narrowing of the capitolunate joint (*black arrows*) in addition to involvement of the radioscaphoid joint. Note preservation of the radiolunate joint (*white arrows*). There is no significant migration of the capitate at this stage

**Fig. 11:**
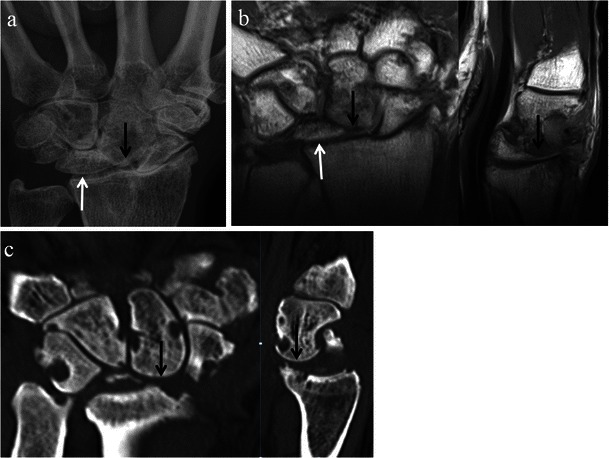
SLAC wrist with late radiological findings. Posteroanterior wrist radiograph (**a**), and coronal and sagittal T1-weighted MRI images (**b**) of the wrist in the same patient, and coronal and sagittal CT reformat images (**c**) of the wrist in a different patient. In addition to extensive osteoarthritic changes, the images demonstrate significant proximal migration of the capitate (*black arrows*) with ulnar displacement of the lunate (*white arrows*)

On imaging, the three stages of SLAC arthropathy can be demonstrated. In stage I SLAC the most radial portion of the radioscaphoid joint is affected first by osteoarthritic changes. The earliest changes seen are sharp spurring at the articular/non-articular junction on the radial side of the scaphoid and at the radial styloid process tip, with loss of the normal rounded curvature of the radial styloid process (Fig. 8). Subsequently, in Stage II SLAC the rest of the radioscaphoid joint is affected with progression to narrowing of the radioscaphoid joint (Fig. 9), followed by Stage III SLAC where there is narrowing of the capitolunate joint (Fig. 10). Ultimately, proximal migration of the capitate with ulnarward displacement of the lunate occurs [8, 14, 15] (Fig. 11).

## Scaphoid non-union advanced collapse (SNAC)

SNAC is due to a non-united fracture of the scaphoid and is a common cause of wrist arthropathy, with a pattern of osteoarthritic change which is very similar to the pattern seen with SLAC wrist. Although SNAC and SLAC are in fact two different derangements, they have a similar pathophysiology and therefore SNAC wrist can be thought of as a variant of SLAC wrist. The difference between these two entities is that as opposed to SLAC where the scapholunate ligament is interrupted with resultant scapholunate diastasis, with SNAC the scaphoid is fractured and the scapholunate ligament and joint are usually preserved. The proximal pole of the fractured scaphoid behaves similar to a small lunate because the fragment is a small spheroid shaped bone which is situated within the spheroidal portion of the scaphoid fossa. This configuration of the non-united scaphoid causes osteoarthritic changes to occur between the radius and the distal scaphoid fracture fragment, progressing proximally but only up to the non-union site [3, 8].

**Fig. 12:**
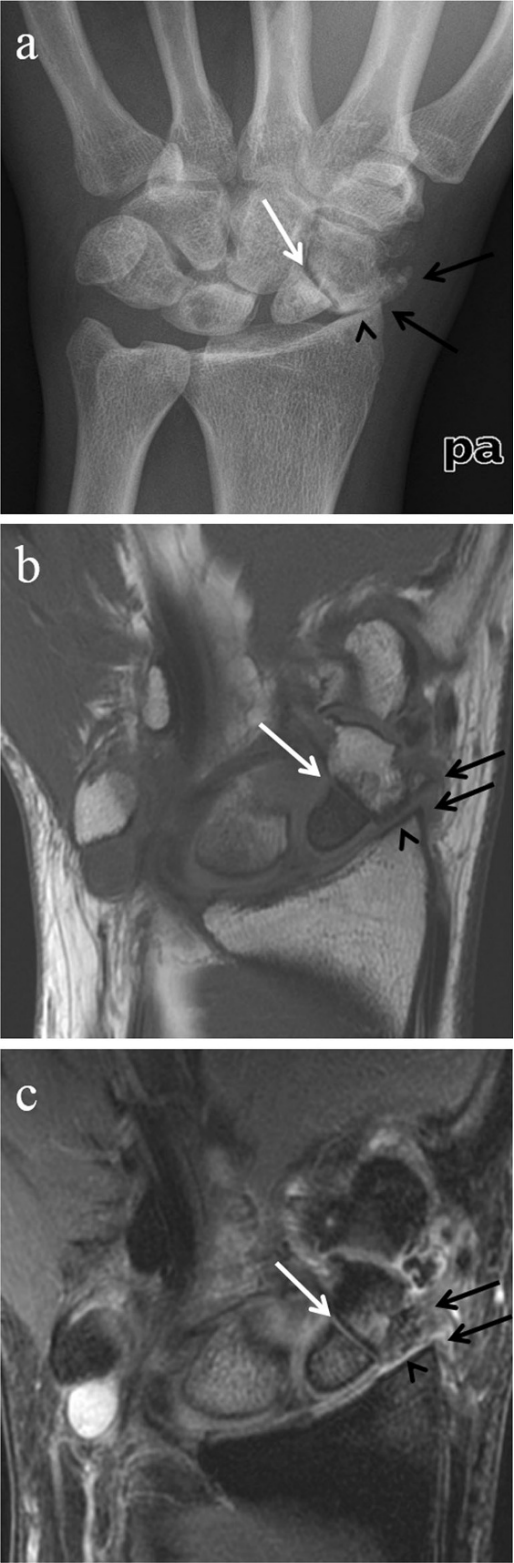
SNAC arthropathy imaging findings. Posteroanterior radiograph (**a**), coronal T1-weighted (**b**) and fat suppressed T2-weighted (**c**) MRI images of the wrist of the same patient showing a non-united fracture of the scaphoid (*white arrows*) with a characteristic SLAC-like type of arthropathy. There is joint space narrowing (*arrowheads*) and osteophyte formation (*black arrows*) at the articulation between the distal scaphoid fragment and the radial styloid. The articular cartilage and joint space between the proximal scaphoid fragment and the radius are preserved. Avascular necrosis of the proximal scaphoid fragment is suggested by its sclerotic appearance on the radiograph, and relatively hypointense signal on MRI

Imaging findings seen with SNAC include arthropathy similar to the imaging findings seen with SLAC. The articulation between the distal non-united fragment of the scaphoid and the distal radius is affected first with joint space narrowing and osteophyte formation. Avascular necrosis of the proximal scaphoid fragment may be evident, although the articulation between the proximal scaphoid fragment and the radius is typically preserved (Fig. 12). Progression to affect the scaphocapitate and capitolunate joints eventually occurs [3].

## Calcium pyrophosphate dehydrate (CPPD) crystal deposition disease

**Fig. 13:**
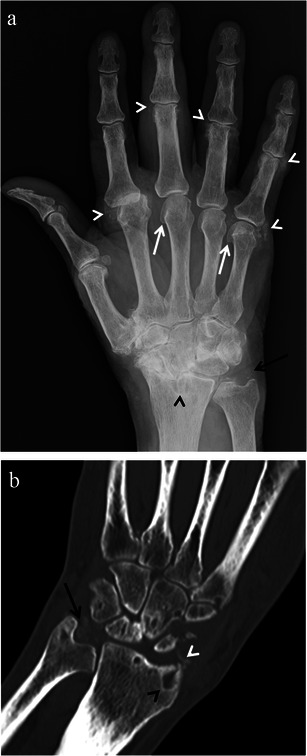
CPPD arthropathy imaging findings. Posteroanterior radiograph of the hand/wrist (**a**) and coronal reformat CT image (**b**) of two different patients demonstrating SLAC wrist and classic findings of CPPD arthropathy. These findings include triangular fibrocartilage (*black arrows*) and peri-articular (*white arrowhead*) calcifications, prominent subchondral cysts (*black arrowheads*) and osteoarthritic changes involving the metacarpophalangeal joints including hook-like osteophytes at the metacarpal heads (*white arrows*). Note is made of significant osteoarthritic changes involving the second metacarpophalangeal joint as well

SLAC wrist is commonly (26 % of the time) seen in patients with CPPD arthropathy [2]. The pathogenesis is pyrophosphate deposition in the interosseous ligaments (scapholunate) leading to ligament laxity and disruption and thus rotational alteration of the scaphoid. Associated findings suggesting CPPD associated SLAC wrist include bilateral involvement, triangular fibrocartilage calcifications, articular and peri-articular soft tissue calcifications, prominent subchondral cysts and osteoarthritic changes involving the metacarpophalangeal joints. Although not entirely specific for CPPD, hook-like or drooping osteophytes at the radial aspects of the metacarpal heads can often be seen [2, 18] (Fig. 13).

## SLAC and SNAC treatment options

**Fig. 14:**
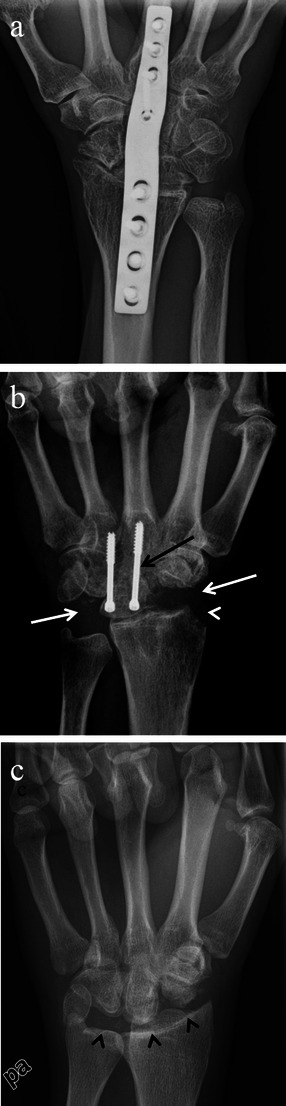
Surgical options for SLAC wrist. Posteroanterior wrist radiographs of three different patients following surgical treatment for SLAC wrist. **a** Complete wrist arthrodesis with a fusion plate is depicted. **b** Patient is status post radial styloidectomy (*white arrowhead*), resection of the scaphoid and triquetrum (*white arrows*), and ankylosis of the lunate and capitate (*black arrow*). **c** Proximal row carpectomy with resection of the scaphoid, lunate and triquetrum (*black arrowheads*) was performed

Asymptomatic SLAC wrist does occur and requires no treatment [19]. For symptomatic SLAC and SNAC arthropathy conservative non-surgical measures are the first line therapy. Non-surgical management includes wrist splinting, oral analgesics and intra-articular steroid injections. When symptoms persist or worsen after conservative measures are attempted, it is then that surgical treatment options are often considered. Surgical options for SLAC and SNAC include arthrodesis which can be complete or partial (four corner arthrodesis or capitolunate arthrodesis), denervation, proximal row carpectomy and radial styloidectomy (Fig. 14). Distal scaphoid pole excision is an additional surgical option which can be used in the treatment of SNAC, and is typically implemented during the early stages of SNAC related arthropathy prior to the development of capitolunate osteoarthritic changes [20, 21]. Also, the presence or absence of avascular necrosis can influence SNAC treatment options. The choice of surgical procedure which is ultimately performed for SLAC and SNAC depends on multiple factors, including both personal surgical preference and the stage of arthritic progression, noting that surgical procedures are commonly combined to achieve optimal results [21].

## Conclusion

SLAC is a frequently encountered wrist arthropathy, with consistent, predictable and progressive imaging features. Along with clinical findings, radiological studies play a vital role in the diagnosis, monitoring and follow-up after treatment of this entity. It is, therefore, crucial to understand the anatomy and function of the scapholunate interosseous ligament, predictable progressive nature of this arthritic disease process, as well as the common causes and treatment options of SLAC wrist.
